# Adaptive Cell-Mediated Immunity in the Mammary Gland of Dairy Ruminants

**DOI:** 10.3389/fvets.2022.854890

**Published:** 2022-04-05

**Authors:** Pascal Rainard, Gilles Foucras, Rodrigo P. Martins

**Affiliations:** ^1^ISP, INRAE, Université de Tours, UMR1282, Nouzilly, France; ^2^IHAP, Université de Toulouse, INRAE, ENVT, Toulouse, France

**Keywords:** mammary gland, mastitis, cell-mediated immunity, vaccine, lymphocytes, hypersensitivity, type 3 immunity, ruminants

## Abstract

Mastitis is one of the greatest issues for the global dairy industry and controlling these infections by vaccination is a long-sought ambition that has remained unfulfilled so far. In fact, gaps in knowledge of cell-mediated immunity in the mammary gland (MG) have hampered progress in the rational design of immunization strategies targeting this organ, as current mastitis vaccines are unable to elicit a strong protective immunity. The objectives of this article are, from a comprehensive and critical review of available literature, to identify what characterizes adaptive immunity in the MG of ruminants, and to derive from this analysis research directions for the design of an optimal vaccination strategy. A peculiarity of the MG of ruminants is that it does not belong to the common mucosal immune system that links the gut immune system to the MG of rodents, swine or humans. Indeed, the MG of ruminants is not seeded by lymphocytes educated in mucosal epithelia of the digestive or respiratory tracts, because the mammary tissue does not express the vascular addressins and chemokines that would allow the homing of memory T cells. However, it is possible to elicit an adaptive immune response in the MG of ruminants by local immunization because the mammary tissue is provided with antigen-presenting cells and is linked to systemic mechanisms. The optimal immune response is obtained by luminal exposure to antigens in a non-lactating MG. The mammary gland can be sensitized to antigens so that a local recall elicits neutrophilic inflammation and enhanced defenses locally, resulting from the activation of resident memory lymphocytes producing IFN-γ and/or IL-17 in the mammary tissue. The rational exploitation of this immunity by vaccination will need a better understanding of MG cell-mediated immunity. The phenotypic and functional characterization of mammary antigen-presenting cells and memory T cells are amongst research priorities. Based on current knowledge, rekindling research on the immune cells that populate the healthy, infected, or immunized MG appears to be a most promising approach to designing efficacious mastitis vaccines.

## Introduction

The MG is an ectodermal appendage giving rise to an epithelial organ the main function of which is to secrete a nutritious liquid food for the offspring. In ruminants, another important function is to provide the newborn, which is agammaglobulinemic at birth, with a crucial humoral immune defense through antibodies to relevant pathogens. As an organ that communicates with the body environment, the MG is exposed to invasion by microbial agents. The lactating MG is prone to infection by bacteria that can thrive in milk and during involution at cessation of milking. We can thus expect that evolutionary pressure has endowed the MG with adapted defenses against infections. Environment-exposed organs such as mucosa, the skin, and exocrine glands are protected from infections by the combined action of the innate and adaptive immune systems. The immune responses that protect epithelial surfaces present specialized characteristics that define the common mucosal immune system (CMIS) (1). Early in the history of research on MG immunity, some basic questions arose: Is the MG a full member of the CMIS and an effector site of this system? Or does it rely on its own local system? Is the MG an inductive site of immunity? What are the relations of the MG with the systemic immune system? Answers to these questions have important implications for our understanding of the response of the MG to infections and for the design and development of vaccines against mastitis. In this review, we endeavored to provide answers or elements of answers to these questions.

Over the last 50 years, a number of articles have dealt with the subject of adaptive immunity in the MG of ruminants. They used a variety of approaches: investigations of natural infections, intramammary inoculation of mammary pathogens, systemic or local immunization regimens, analysis of the humoral response and less frequently of the cellular response, or phenotypic and functional characterization of the cells in MG secretion or tissue. A synthesis of these sources of information is not easy due to the disparity of experimental approaches, the constant evolution of knowledge and concepts in a fast-evolving scientific field, sometimes contradictory results, and our limited knowledge of mammary immunity. Despite these shortcomings, it is possible to identify some clear features characterizing adaptive immunity in the MG of ruminants. These characteristics, interpreted and assembled according to current concepts in adaptive immunology, allowed us to propose a schematic vision of acquired immunity to MG infections. However, we did not address in this review the interactions of the adaptive immunity with the innate immune system. In particular, the interactions of lymphocytes with mammary epithelial cells have not been considered, although these interactions are crucial for the inductive and effector arms of cell-mediated immunity. Therefore, we have deliberately focused on immune responses dependent on memory T cells.

Historically, acquired MG immunity has been considered as a manifestation of delayed hypersensitivity, and it was realized that its effects could either ameliorate or aggravate the outcome of infection. Early on, the role of lymphocytes rather than antibodies was established in the induction of milk leukocytosis (2, 3). Cell-mediated immunity has long been known to play a significant role in the pathogenesis of mastitis. A number of observations and experiments support this notion. Cows infected with *S. aureus* showed an increased reactivity of the MG upon repeated infections (4). A primary infection of the bovine MG with *Mycoplasma dispar* induced an enhanced influx of neutrophils into milk at the onset of a subsequent reinfection (5). This enhanced milk leukocytosis can be induced by parenteral immunization with bacterial antigens. For example, the magnitude of the milk leukocytosis induced by intramammary infusion of killed staphylococci increased with the extent of previous experience with staphylococcal components, such as a previous parenteral immunization of cows with killed staphylococci (6). The kinetics of the inflammatory response to inoculation of the MG with *Streptococcus agalactiae* was accelerated by sensitization to a streptococcal protein antigen by subcutaneous immunization (7). From these observations, it appears that mastitis is conditioned by cell-mediated immunity. How this happens and how this immunity can be harnessed to protect the MG through vaccination are major research topics for improving mastitis control.

## The Mammary Gland as an Immune Inductive Site

### It Is Possible to Elicit a Local Immune Response in the MG

Historically, one of the main drivers for exploring the MG capacity to mount an immune response has been the need to improve the efficacy of vaccines against mastitis. Early attempts have been directed to the production of antibodies. Pioneering studies have been carried out with ewes by Australian researchers. They focused their work on the local production of antibodies elicited by the intramammary infusion of antigens. They convincingly demonstrated that a local antibody response can be induced in the MG by intraluminal infusion of antigens. The best results were obtained when antigens were administered several weeks before parturition, which did not depress milk yield during the subsequent lactation (8, 9). The authors supposed that in the weeks following involution after cessation of milking, the influx of leukocytes equips the MG with the cells necessary to mount a local immune response. At involution, the MG is invaded by lymphoid cells whereas during lactation, very few lymphocytes can be seen in the mammary tissue. The physical presentation of antigen seemed to make a difference: *Salmonella* flagellin in polymeric particulate form induced a much greater local response (production of IgA) than infusion with the soluble monomer (10). Whole killed bacteria such as *Brucella abortus* induced high local IgA responses (11). During lactation, particulate antigens (killed *Brucella*) may not be able to induce antibodies because, contrary to soluble antigens (staphylococcal toxoids), they are eliminated during the milking that follows the infusion (12). In a study comparing the effect of four immunization schedules of cows on the production of antibodies (IgA and IgM) in milk, the combination of systemic immunization with adjuvant during MG involution followed by local antigen infusion was the most effective regimen (13). Systemic immunization at drying-off would allow antigen-specific lymphocytes to seed the MG. In some cases, local intramammary immunization with bacterial antigens induced antibody titers higher in milk than in serum. Antibody titers and activities were consistently higher in the immunized gland than in the control unimmunized glands of ewes and cows (9, 14). From these experiments, we can conclude that it is possible to elicit a local antibody response in the MG, in particular in non-lactating glands and with particulate antigens. However, it has been hypothesized that, in view of the lack of lymphoid tissue associated with the MG and capable of responding to locally administered antigen, the precursor cells producing IgA antibodies arise in mucosal tissues distant from the MG, such as the oral cavity or the gut and respiratory tracts, before migration to the mammary tissue (15).

### The MG of Dairy Ruminants Does Not Belong to the CMIS

Once the possibility of inducing a local immune response in the MG was established, the question arose of the relationship between the MG and the mucosal and systemic immune systems. A fundamental observation was that the reactivity repertoire of lymphocytes isolated from human milk differs from the repertoire of blood lymphocytes (16). In particular, milk lymphocytes and antibodies react to enteric pathogens, representing an immunity that plays an important role for the passive protection of the neonate. This observation, as well as other studies carried out in laboratory rodents, led to the notion of compartmentalization of the immune system, and to the recognition of the existence of a network of immune responses linking different mucosal surfaces exposed to microbiota, such as the enteric, oral, nasopharyngeal, and respiratory mucosae. This common mucosal-associated lymphoid tissue (MALT) links two components: the true mucosae that are inductive sites of immunity for the whole MALT, and secretory glands and organs that are not directly stimulated by mucosal antigens, such as the salivary and mammary glands, or the urinary tract (1). From this perspective, the exocrine glands can mount a local immune response, but this response does not generalize to the MALT. According to Parmely and Beer (17), the gut-associated lymphoid tissue (GALT) and bronchial-associated lymphoid tissue (BALT) are the sites where T and B cells that will seed the MG are generated. The gut and the upper airways possess lymphoid structures that allow T and B cells to be educated and humoral and cell-mediated immunity to be elicited. Regarding humoral immunity, the induced cells are IgA plasmablasts. These two mucosae have the capacity to generate immune cells that can migrate to their initial location but also to other organs of the MALT, including the MG and salivary glands. On the contrary, the mammary and salivary glands do not have the capacity to generate immune cells able to migrate to the CMIS. This statement is based on observations and experiments performed on rodents, swine and humans. However, does it apply to ruminants?

In fact, a number of arguments contradict the view that the MG of ruminants belongs to the common MALT. Contrary to what occurs in rodents and humans, IgG_1_ antibodies, not secretory IgA, are the major immunoglobulins in the colostrum and milk of ruminants (9, 18). The intraperitoneal immunization of ewes with ovalbumin elicits IgA antibody-containing B cells in the intestine, but not in the MG, an observation that does not support a relocation of IgA plasma cell precursors from the GALT to the MG (19). The combination of intraperitoneal and intramammary immunization has been shown to result in higher numbers of IgG_1_ antibody-producing cells in mammary tissue than intramammary immunization only (19). This enhanced IgG_1_-specific response was not reduced, and even increased when the mesenteric or mammary lymph nodes (LNs) were removed prior to immunization (20). The authors concluded that the population of the MG by antigen-specific plasma cells following peritoneal immunization does not result from the seeding by precursor cells originating from GALT or mammary LNs. The results suggest that the plasma cell precursors could be generated in the LNs of the systemic immune system. Moreover, most of IgA antibodies in the milk of ruminants are serum-derived during lactation, not produced locally by plasma cells (21). There was no evidence for any substantial translocation of IgA precursor cells from GALT to the MG at any stage of lactation (20). Most of the IgG_1_ in colostrum and milk are transferred from the blood (9, 22). Relevant information was obtained by immunizing cows a few weeks before parturition with T4 bacteriophages by different routes: intramammary (intraductal) into one quarter, intestinal (through a jejunum fistula), subcutaneous near the right superficial cervical LN or the superficial inguinal (mammary) LN (23). Neither IgA nor IgG_1_ antibodies nor plaque-forming cells were induced in MG secretions from preparturient cows following intestinal immunization, contrary to the intramammary route. The responses to superficial cervical and mammary LN immunizations were similar regarding the presence of IgA and IgG_1_ plaque-forming cells in MG secretions but much less intense than the response induced by the intramammary route. The authors inferred from these results that in cattle there is no gut-mammary axis for the seeding of the MG with intestinal lymphoblasts. Another study supports this view. Radiolabeled autologous lymphocytes prepared from the mammary or ileal mesenteric LNs were re-injected into dairy cows (24). A higher percent of radiolabeled mammary LN cells was found in the mammary, superficial cervical, and bronchial LNs of non-lactating heifers or lactating cows than in the ileal and jejunal mesenteric LNs. Conversely, more radiolabeled ileal mesenteric cells migrated to the jejunal, ileal and bronchial LNs than to the mammary and superficial cervical nodes. These data support the hypothesis that lymphocytes do not migrate efficiently between the gut and the udder, i.e., that the entero-mammary link is poorly functional in the cow and in the ewe (25), contrary to the sow (26). We can conclude that, as a secretory gland, the MG of ruminants is not an inductive site of the common MALT, and as a feature of ruminants, its immunity is not dependent on the gut-associated lymphoid tissue.

### The MG of Ruminants Does Not Express the Addressins of the Mucosal Lymphoid System

An important issue is that of the homing of lymphocytes to the MG and the resulting seeding of the MG tissue with antigen-specific cells. Once naïve lymphocytes have encountered their cognate antigen presented by APCs and are instructed to differentiate into effector cells, they engage in a few cycles of multiplication and migrate to the inflammatory site of bacterial intrusion. After they have exercised their functions, most die by apoptosis, and only a few precursor cells become memory lymphocytes. It is these cells that will be responsible for the immune memory of the adaptive response. There are several types of long-lived memory cells that follow distinct trafficking patterns (27). Circulating memory T cells (Tcirm) are a heterogeneous population of memory cells that circulate between the blood and lymphoid organs, and can be mobilized into non-lymphoid tissues. Resident memory T cells (Trm) are retained in the tissue, where they can rapidly react to local antigen re-exposure, but can occasionally recirculate while keeping a predilection for their tissue of origin (28). The location of T cell priming dictates homing and differentiation of naïve T lymphocytes, and eventually the tissue tropism of memory cells (29). This has been established for the homing of T cells to the skin, intestine, or respiratory tract. According to the multistep model of leukocyte migration, tissue tropism is determined by the combined interactions of lymphocyte integrins and chemokine receptors with tissue-specific adhesion molecules including vascular addressins and chemokines (Table 1). For example, T cells activated in the GALT upregulate the α4β7 integrin and the CCR9 chemokine receptor, to interact with the mucosal addressin cell adhesion molecule-1 (MadCAM-1) and the chemokine CCL25 which is constitutively produced by small intestine epithelial cells (30). In the MG of porcine, murine and human species, high levels of CCL28 mRNA, also named mucosae-associated epithelial chemokine (MEC), are produced by epithelial cells, and the corresponding receptor CCR10 is expressed by mammary T lymphocytes and IgA plasma cells (31–33). In the pig and the mouse, the recruitment of IgA plasmablasts to the MG depends on the expression of vascular cell-adhesion molecule-1 (VCAM-1)/α4β1 and MadCAM-1/α4β7 in conjunction with CCL28/CCR10 (33). CCL25 also is likely to contribute to the recruitment of T cells in the MG in swine (34). These results are compatible with a functional link between the MG and the gut and upper respiratory tracts (33).

**Table 1:** Homing determinants: integrins/ligands, vascular addressins and receptors.

**Lymphocyte homing molecule**	**Distribution**	**Ligands**	**Ligand expression**
L-selectin, CD62L, LAM-1	Naïve T cells, Tcm	Glycoproteins with sialyl Lexis X, PNAd	Endothelium, HEV of peripheral LN
αLβ2, LFA-1, CD11a/CD18	Lymphocytes, granulocytes, monocytes, macrophages	ICAM-1, 2, 3, 5	Endothelium, DCs
α4β1, VLA-4, CD49d/CD29	Lymphocytes, granulocytes Dendritic cells	VCAM-1, MadCAM-1, fibronectin	Endothelium, extracellular matrix
α4β7, LPAM-1, CD49d/ITGB7	Mucosal lymphocytes, dendritic cells	MadCAM-1, VCAM-1, fibronectin	HEV of mucosal tissues, extracellular matrix
αEβ7, HML-1, CD103/ITGB7	Intra-epithelial lymphocytes	E-cadherin	epithelium
CD44, Hermes, Pgp-1	Tem cells	Hyaluronic acid	
E-cadherin	Epithelial cells, leucocytes	E-cadherin	epithelium
PNAd	HEV	L-selectin	Leucocytes

*ICAM-1, intercellular adhesion molecule-1; LAM-1, leucocyte adhesion molecule; LFA-1, lymphocyte function associated antigen-1; LPAM-1, Lymphocyte Peyer patches-adhesion molecule; MadCAM-1, mucosal addressin cell adhesion molecule; PNAd, peripheral node addressin; PSGL-1, P-selectin glycoprotein-1; VCAM-1, Vascular cell-adhesion molecule-1; Tcm, central memory T cells, Tem, effector memory T cells; DCs, dendritic cells; HEV, high endothelial venules*.

Do these findings apply to the MG of dairy ruminants? Few studies examined the presence of addressins and homing receptors in the tissue and cells of the MG in the cow, ewe, and goat. It has been reported that the vascular addressin α4β7, also known as the lymphocyte Peyer's patch-specific homing receptor LPAM-1, was expressed on a low percentage of all lymphocyte subsets at all-time points before and after experimental infections of dairy cows (35). The expression of addressins was investigated in the bovine MG by immunohistochemistry and RT-PCR analyses at four different physiological stages: pregnancy, colostrum formation, lactation, and involution (36). MadCAM-1 was not detected in mammary tissue at any physiological stage. VCAM-1 was found in alveolar tissues during the colostral and lactation phases. VCAM-1 expression was found on both large and small venules in the mammary LN. Peripheral node addressin (PNAd) was detected in the mammary LN at all physiological stages, but it was not found in mammary alveolar tissue. No lymphocyte expressed the integrin component β7 in alveolar mammary tissues. CD62L (L-selectin) staining of lymphocytes was detected in mammary LN, but not in mammary tissue due to non-specific staining. However, L-selectin, a peripheral lymphocyte homing receptor (37), was found on MG lymphocytes (38). The local immunization of the MG during the dry period with *Candida albicans* induced the local production of IgA and an increase in the number of lymphocytes in the mammary alveolar tissue, but no lymphocyte expressed the integrin component β7 (39). There was no detection of MadCAM-1 in mammary tissue of immunized quarters, and the mRNA expression was not detected by RT-PCR. There was no staining of small venules for VCAM-1 or PNAd in alveolar tissue. In a flow cytometry analysis of milk lymphocytes of cows at different stages of paratuberculosis, a small proportion of lymphocytes (7–14%) were LPAM-1^pos^ (lymphocyte Peyer patches-adhesion molecule-1), 85% were CD44^pos^ and 80% LFA-1^pos^ (40). In conclusion, these studies did not replicate the findings obtained with mouse or sow MGs. The MG of dairy ruminants does not share the lymphocyte homing characteristics of mucosal organs. It may not be surprising that the major draining LNs, the mammary (superficial inguinal) LNs, which drains both the MG and the regional skin, connective and muscle tissues, has been found to express the PNAd and VCAM-1 vascular addressins. These LNs cannot be considered as LNs of the MALT, as are the mesenteric LNs. The LNs draining the MG are part of the peripheral lymphoid system, which means that the MG immune system is linked to the systemic immune system. This peculiarity of the MG of ruminants is a matter of speculation.

Ruminants harbor a large stomach reservoir fulfilled with huge numbers of potentially-pathogenic bacteria. Segmented filamentous bacteria were the first commensal micro-organism known to drive the differentiation of Th17 cells in the mouse intestine, and other bacteria attached to intestinal epithelial cells have similar priming capacity (41). The absence of an entero-mammary axis in cattle and ruminants, and the relative independence of the MG to the CMIS might be an evolutionary way found by these species to avoid the recruitment into the mammary tissue of potentially harmful lymphocytes that, if in excess, have a damaging inflammatory potential upon exposure to colonizing fecal bacteria.

It appears that we know little about the combination of homing molecules, selectins, integrins, vascular addressins, chemokine and chemokine receptors on T cells and mammary tissue that dictates the homing of T cells to the MG of ruminants. What are the tissue factors that condition effector and memory T cell differentiation *in situ*? We also do not know much about the MG-specific “area code” that determines the interaction of T cells with the mammary tissue. This ignorance has negative implications on our capacity to devise efficient vaccination protocols.

### The Question of Antigen Presentation in the Mammary Gland

A few studies show that antigen-presenting cells (APCs) are present in the MG epithelium, suggesting that the MG provides a suitable environment to initiate an immune response, in particular during the dry period. Many stellate or spindle-shaped MHC II^pos^ cells have been identified in the basal region, in close apposition to or within the ductal epithelium of non-lactating ewes (42). Numerous MHC class II^pos^ cells were found interspersed between the alveolar and ductal epithelial cells of fully involuted MG of ewes (43). In all quarters of dry cows examined, MHC class II^pos^ cells were seen in the inter-alveolar and inter-lobular connective tissue (44). Most of the cells had the morphological appearance of macrophages. Macrophages (ionized calcium binding adaptor molecule 1 IBA1^pos^ cells) have been found in the ductal epithelium of pre-pregnancy lambs with increased density with age (45). Infusion of formalin-killed *S. uberis* increased the cellular expression of class II antigen both in connective tissue and in the epithelium lining the ducts and particularly the gland cistern. APCs have been found in the epithelium of the teat sinus, being most numerous at the Fürstenberg's rosette (46). At this site, MHC class II^pos^ cells were present in the uppermost layer of the epithelial bilayer and between the two layers, in higher number in dry than in lactating glands. Another cell type (CD11c^pos^ CD205^pos^), possibly dendritic cells, was present almost exclusively in the bilayer epithelium at the level of the Fürstenberg's rosette. DCs identified based on morphology, on low expression of CD14, and high expression of MHC II and CD11c or CD205 were found in alveolar and ductal epithelia and in the connective tissue (47). Scarce in the alveoli, these DCs were more frequent and regularly spaced in the ductal epithelium. Macrophages identified based on MHC II expression along with high expression of CD14 and CD11c were also found, and were globally at least two fold more numerous than DCs in the mammary tissue. These two cell types were also found in the mammary LNs. Recently, a category of APCs, tissue-resident ductal macrophages, has been described in the MG of virgin, pregnant and lactating mice based on high expression of MHC-II, CD11c and CX3CR1 (48, 49). Two categories of macrophages constituted the most numerous immune cells: stromal macrophages and “ductal macrophages”, the latter located between epithelial and myoepithelial cells. The epithelium-associated macrophages have a stellar shape, are distributed in alveoli and ducts, and showed transcriptome changes through the reproduction cycle. They scavenge apoptotic epithelial cells during involution, but their contribution to the immune defense of the MG against infections was not investigated, although they express anti-inflammatory (IL-10) and Th17-related (IL-23, IL-22 an IL-17A) modulatory pathways during lactation (48).

It is therefore likely that the MG is equipped to sample antigens from the cisternal, ductal, or alveolar lumen by specialized APCs and to transfer the loaded cells to the draining LNs, although this has not been formally demonstrated. The presence of APCs, together with the local inflammatory response triggered by infection, fulfills the conditions for the initiation of an immune response. This is supported by studies showing consistently that immune responses are induced by intramammary immunizations. Of note, there are two ways the MG immune system can be stimulated, resulting in different immune responses. Infusion of killed *Brucella* into the lactiferous sinuses (**transepithelial antigen exposure**) of dry MGs in ewes induced higher titers of antibodies in milk than the injection of bacteria into the mammary tissue (**interstitial antigen exposure**) near the mammary LN, although titers of circulating antibodies were similar (50). Moreover, luminal infusion induced more IgA antibodies, whereas tissue injection induced more IgG_2_ and a local granuloma. The author concluded that the interstitial presentation of antigen probably did not induce local synthesis of antibodies, contrary to the transepithelial presentation. The different immune responses may result from the presentation of the antigen by different types of APCs (intra-epithelial vs. connective locations) or APCs in a different state of maturation. Antigen processing by epithelium-associated APCs (dendritic cells or macrophages) likely differs from the handling by APCs like inflammatory monocytes attracted by the local inflammation caused by the injection of a vaccine in the mammary tissue. From this perspective, the APCs conditioned by the epithelium microenvironment would instruct the naïve T lymphocytes in a way characteristic of the transepithelial presentation of antigen. Transepithelial antigen exposure would induce both a systemic and local immune response, while interstitial antigen exposure would mainly induce a systemic response.

Another feature of the luminal infusion of antigen is that it elicits an immune response locally restricted in that infusion of antigen into one gland does not result in the same generalized response to all udder glands. Several studies have shown that antibody titers are higher in infused glands than in uninfused ones. Intramammary immunization of cows with *E. coli* during the dry period elicited antibodies in colostrum and milk (14). Killed *Brucella* infused into the dry MG of ewes induced antibody titers in milk mainly in the infused half udder, in particular agglutinating antibodies, supposed to be IgA and IgM and to be produced locally (50). When the MG of pregnant ewes was infused with killed *Salmonella* in one gland and killed *Brucella* in the other, antibody titers were much higher in the milk of the immunized gland than in the milk of the contralateral gland for each antigen (51). The combination of peritoneal followed by local (intramammary in one half udder) immunization with ovalbumin generated antigen-specific plasma cells only into the immunized gland, not in the contralateral gland (20). This supports that there is no “common MG mucosal immune system” in dairy ruminants. It should be noted that the lymphatic drainage of the two sides of the MG is separate. Thus, a regional system (the glands and their draining LNs) might operate to generate the local response. This is confirmed by the observation that when antigen (inactivated streptococci) is injected into the right mammary LN, antibodies appear first in the right quarters (52). However, the mammary lymphadenectomy experiment mentioned above (20), which resulted in a higher local immune response, indicates that mammary LNs are dispensable. It is likely that upon their stimulation and handling of antigen, the APCs in the MG migrate to the regional draining LNs, the mammary (superficial inguinal) LN, the deep inguinal LN and the sacral and medial iliac LNs, and probably other LNs of the systemic immune system, where they instruct antigen-specific (naïve) lymphocytes. Then, the antigen-experienced lymphocytes may circulate and home to different sites and tissues, including the MG. In this hypothesis, a systemic immunization has the same potential as a local intramammary immunization into one gland to share the response to all mammary glands. We have seen that this does not hold true for the immunized gland, which strongly suggests that a local pathway is superimposed on the systemic one to explain the original restriction of local immunization.

Tissue diffusion and uptake of soluble and particulate antigens by the MG of dry cows has been studied by infusing a soluble antigen (high amount, 50 mg or 1 g ovalbumin) into a quarter and particulate antigen (large number of killed *S. uberis*, 10^10^ CFU) into another quarter, before collection of mammary tissue and draining LNs for evaluation of antigen retrieval (53). Ovalbumin reached the mammary LNs very early (1 h after infusion) and was also found in blood and in non-infused quarters. Ovalbumin was found free in the tissue (paracellular staining) or as small or large aggregates in the cytoplasm of a proportion of cells of the luminal layer of the epithelium of the teat. Ovalbumin was also found in the connective tissue and lymph vessels. It appeared that the dry MG was quite permeable to low molecular weight soluble antigen, allowing a wide diffusion of the antigen to the systemic immune system and, remarkably, to uninfused quarters. In comparison, few *S. uberis* were found in the tissue and in the draining LNs 3 to 6 h after infusion. Cocci were found within the cytoplasm of epithelial cells of mammary ducts, but also in the connective tissue without evidence of phagocytosis. It thus seems that antigens can reach the draining LNs without handling by mammary APCs. Of note, ovalbumin stimulated a systemic antibody response (small IgG_1_ and IgG_2_ response) but no local response was detected whereas bacteria induced mainly a local antibody response (54). This would suggest that a certain degree of local (mammary) handling of antigen is necessary to induce the local immune response, along with some inflammation induced by microbe-associated molecular patterns (MAMPs). The free diffusion of soluble antigens may contribute to better efficiency of intramammary immunization during the dry period, when epithelium tight junctions are leaky, than during the lactating period, when tight junctions are sealed (55). This is supported by the observation that fewer lymphocytes are retained in the draining inguinal LNs of lactating mice compared to fully involuted or nulliparous mice after intramammary infusion of ovalbumin, indicating reduced presentation of antigen by dendritic cells (56).

**Figure 1:**
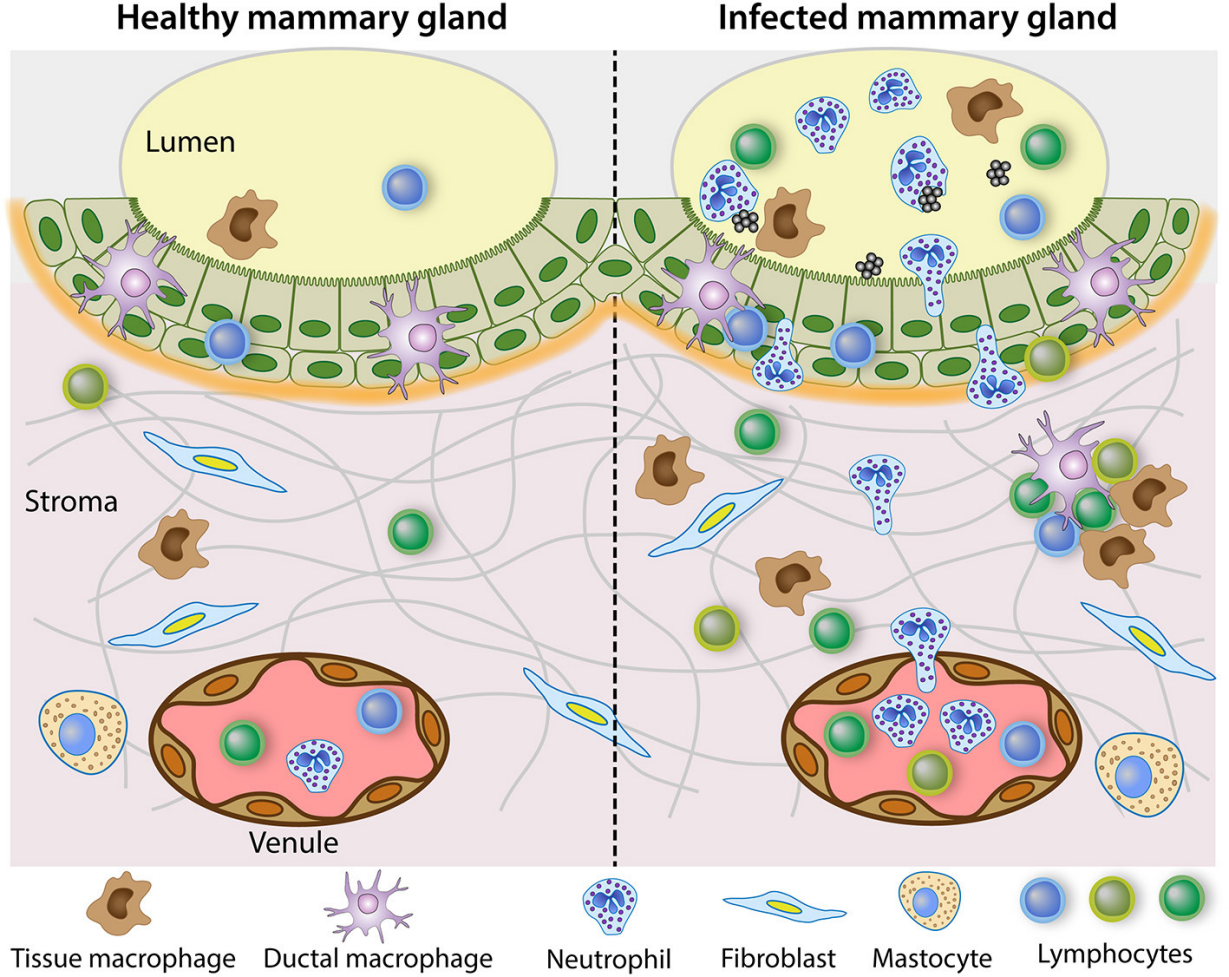
Steady state immune system in the healthy mammary gland and reactive leukocytosis when infection occurs. The healthy lactating mammary gland is relatively poor in leucocytes, in the lumen but also in the tissue. The mammary epithelium schematized in the figure is the bilayer epithelium of the cisterns and large ducts. It is populated with ductal macrophages and a few lymphocytes, mainly CD8^pos^ T cells. In the sub-epithelial stroma, which is richly vascularized, a few lymphocytes, mainly CD4^pos^ T cells are scattered along with stromal macrophages. By contrast, the infected mammary gland recruits inflammatory cells, mainly neutrophilic granulocytes, and attracts monocytes and multiple types of lymphocytes. In chronic infections, lymphocytes aggregates and inducible tertiary lymphoid formations may develop.

In healthy MGs, lymphocytes, macrophages and DCs are distributed diffusively throughout the epithelium, in subepithelial lamina propria and in the connective tissue. Leukocytes are most numerous in the stroma of the Fürstenberg's rosette at the proximal end of the teat canal, but they usually do not show organized lymphoid formations. However, there have been reports where the accumulated leukocytes have taken the appearance of germinal centers (57, 58). In the folds of the rosette, accumulations of leukocytes, mainly IgG_1_ plasma cells, sometimes resembling germinal centers, were found mainly in infected glands of lactating or dry cows. Lymphoid aggregates described as teat nodules have been found at the border between teat duct and teat cistern of ewes naturally infected or not (59). These lymphoid cell aggregates, sometimes lymphoid nodules with germinal center, were more frequent in case of concomitant infection (60). The lymphoid cell formations were induced at the junction of the teat canal and cistern by the introduction of bacteria (*Mannheimia haemolytica*) in the teat canal of lactating ewes (61). Immunohistochemical analysis revealed an influx of CD3^pos^ cells, a few γδ T cells and CD8^pos^ cells, but no CD4^pos^ cells, in the lamina propria between the teat canal and cistern. Lymphoid-like structure with CD3^pos^ cells could be seen at the periphery of a lymphoid follicle-like structure. These observations are reminiscent of the formation of **inducible tertiary lymphoid structures (TLS)**. These structures, also known as ectopic lymphoid tissue, are induced in non-lymphoid organs by lasting infections and play a role in local immunity (62, 63). They present a structured cellular architecture with separate B and T cell areas, B cell follicles with germinal centers, follicular dendritic cells, high endothelial venules (HEV) that express relatively high level of vascular adhesion molecules (addressins), **PNAd** complementary receptor for L-selectin and VCAM-1, lymphocyte homing depending on L-selectin/PNAd, α4β1/VCAM and LFA-1 migration pathways (63). The activation of PRR by microbial components could directly activate innate immune cells involved in the formation of ectopic lymphoid tissue (64). TLS might comprise inductive sites for immune responses and permit naïve lymphocyte trafficking. Currently the information about the tertiary lymphoid structures in the MG is limited. Their presence is reported at the distal end of the teat cistern, and they seem mainly related to humoral immunity. In the experiment aiming at exploring the fate of antigens in the MG, the bilayer epithelium at the level of the Furstenberg's rosette did not take up ovalbumin or *S. uberis*, and the antigens were not found in the follicles of the rosette folds, whereas the antigens were found in some cells of the outer layer of the teat cistern epithelium (53). The role of the rosette formation in the defense of the MG remains to be defined. Intriguingly, lymphocyte aggregates resembling tertiary lymphoid follicles have been found in the MG of neonatal and 5–9.5 month-old lambs (45). These formations in the stroma adjacent to the mammary epithelium exhibit the typical arrangement of central B cells surrounded by T cells, in association with high endothelial venules expressing the vascular addressin PNAd. The presence of tertiary lymphoid structures in the absence of previous lactation, exposure to antigen, infection or inflammation (no neutrophilic infiltration) suggests that this is a normal feature of MG development. Further characterization of these formations to delineate their role in the immune defense of the MG is needed. Besides TLS, which are associated with HEVs, lymphocyte aggregates, which do not have HEVs and comprise only antigen-experienced lymphocytes, have been characterized in non-lymphoid tissues under various infectious or inflammatory conditions (65, 66). Such aggregates have also been described in the MG of ruminants. A scheme depicting the main mediators of cellular immunity in healthy and infected MG is shown in Figure 1.

## The Mammary Gland as an Immune Effector Site of Adaptive Cell-Mediated Immunity

### Lymphocyte Subsets in the Secretions From Healthy Glands

Isolation of lymphocytes from the abundant mammary gland secretion of dairy ruminants is rather easy, and their characterization can provide indications on the capacity of the MG to mount an immune response. As a result, many studies are available on the phenotype of milk lymphocytes. However, leucocytes are not abundant (usually <50,000/mL) in the milk from healthy MGs. Their numbers are higher in foremilk than in other fractions (67). The proportions of cell types differ as a function of the milking fraction: there are more macrophages in cisternal milk, more neutrophils in alveolar fractions, but the proportion of lymphocytes is rather constant (68, 69).

The relative proportions of lymphocyte subsets vary in secretions from healthy MGs depending on the lactation stage. Most studies agree that in dry secretions the CD4/CD8 ratio is > 1, as in blood, but that this ratio changes dramatically at parturition to <1 during lactation (70–76). The same result applies to ewes (77). The proportion of CD4^pos^ T cells in milk may increase with parity (78). In milk from heathy glands, TCRαβ CD3^pos^ CD8^pos^ T cells (cytotoxic/suppressor T cells) are the most numerous, followed by CD3^pos^ CD4^pos^ helper T cells. Most of these cells have a memory phenotype (CD45RO+) and a majority an activated phenotype (CD2^pos^) (71, 73, 79).

In cattle, γδ T cells comprise two subsets that can be subdivided based on cell-surface phenotype (80): WC1^pos^ cells that are CD8^neg^ and CD2^neg^ and WC1^neg^ cells that express CD8 αβ heterodimer and CD2 (81). These two subsets tend to have tissue-specific functions, consistent with a role in inflammation for WC1^pos^ cells and a role of sentinel mucosal cells for WC1^neg^ γδ T cells (82–84). Accordingly, few WC1^pos^ γδ T cells were found in milk and dry secretions (73, 76, 79). However, sizeable proportions of γδ T cells have been found at calving (75, 78).

Relatively few B cells have been found in milk throughout lactation (71, 73, 79) and in the secretions of dry glands (85). Less than 5% of milk lymphocytes were CD21^pos^ MHC II^pos^ B cells (73).

There is a marked increase in cell concentration in MG secretion during the first week after cessation of milking (up to 10^7^ cells/mL). In uninfected quarters, the proportion of neutrophils is initially as high as the proportion of macrophages, then macrophages predominate and lymphocytes represent one third of the cells in the secretion of involuted glands (86). In MG cistern during the dry period, most of the leukocytes are lymphocytes, with a majority of T cells over B cells, and CD4 over CD8 T cells (87). In the mid-dry period, lymphocyte proportion dominates over macrophages (88).

It has long been realized that although lymphocytes in mammary secretions have gained attention as vectors of cell-mediated immunity, their activity may not truly reflect the contribution of lymphocytes to MG defenses (89). It is likely that events taking place in the subepithelial connective tissue are of higher importance to the orchestration of the immune response than those that occur in the gland lumen. Interactions between cells through cell-cell contact and diffusion of their soluble mediators is much more efficient in the tissue than dispersed and diluted in a liquid that is shed at each milking. This is supported by the observation that a much greater proportion of lymphocytes is seen in the subepithelial connective tissue and in close association with the epithelium lining than in the MG lumen during infection or antigen-specific inflammation (90, 91). Moreover, in the milk of healthy MGs, cell numbers are low, and the cells can hardly be considered as resident cells as most of them are removed from the MG at each milking.

### Recruitment of Lymphocytes Under the Regime of Inflammation

The recruitment of leukocytes into tissues is conditioned by signals from the inflamed endothelium [reviewed in (92)]. Antigen-experienced T cells are poised to migrate to sites of inflammation. The innate immune response to bacteria releasing MAMPs generates inflammatory cytokines and chemokines that induce upregulation of vascular adhesion molecules, such as VCAM-1, recognized by the integrin α4β1, and the presentation of chemokines by endothelial cells. Upon stimulation with MAMPs, mammary epithelial cells produce an array of chemokines such as CXCL8, CCL2, CCL5, CXCL10, and CCL20 that can attract a variety of leukocytes and antigen-presenting cells (93, 94).

In the course of natural intramammary infections of cows by *E. coli* or streptococci, CD4^pos^ T cells became the predominant T cells in milk from infected glands (95). The numbers of recruited lymphocytes increased (both CD4^pos^ and CD8^pos^ cells), more in samples from *E. coli* infection than in samples from streptococcal infections. By RT-PCR analysis on whole cell population *IL6* and *IFNG* mRNA were found in all samples (healthy and infected), but not *IL2* or *IL4* mRNA. Another study of naturally occurring mastitis showed that αβ and γδ T lymphocytes were recruited in milk along with the dominant population of neutrophils (38). The CD4/CD8 ratio changed from 0.68 in normal milk to 1.39 in milk of staphylococcal mastitis but remained at 0.65 in milk of streptococcal mastitis. The authors concluded that different αβ and γδ T-cell subsets are recruited to the udder, depending on the mastitis pathogen and the host immune status. There was an increase in γδ /αβ T cell ratio in mastitis milk. Most γδ T cells in milk were CD8^neg^, and about one third were CD2^pos^. L-selectin expression was reduced on milk lymphocytes and neutrophils, consistent with previous studies that showed that L-selectin is shed during exudation. CD18 was upregulated on lymphocytes and neutrophils (marker of activation) from the blood and milk of cows with mastitis compared to cells from blood and milk of healthy cows. After infusion of *E. coli* into the MG of lactating cows, there was an early influx of CD8^pos^ T cells, followed by an increase in CD4^pos^ and B cells (96). Following experimental infection with *S. uberis*, the increase in somatic cells count (SCC) started at 30 hpi and peaked at 48 hpi, mainly with neutrophils (97). Influx of CD3^pos^ cells was minimal at 48 hpi, but high at 96 hpi with a majority of CD4^pos^ cells over CD8^pos^ cells. Later on, the influx of CD8^pos^ cells increased so that by 312 hpi they predominated over CD4^pos^ cells. γδ T cell numbers increased after 96 hpi but remained in the minority throughout the follow-up period. Infection of MG with *Streptococcus dysgalactiae* was associated with an increase in the proportion of CD3^pos^ T cells, CD4^pos^, CD8^pos^, and CD4^neg^-CD8^neg^, but hardly of CD21^pos^ B cells (98). Among T cells, the most numerous were CD8^pos^, then CD4^neg^-CD8^neg^ (likely γδ T cells), then CD4^pos^. In milk samples with elevated SCC, the proportion of CD8^pos^ increased, and also the proportion of CD4^neg^-CD8^neg^ (supposed γδ T cells) (99). The proportion of CD4^pos^ cells also increased, but the CD4/CD8 ratio remained <1. The ratio was highest in “no-growth” milk samples (elevated SCC with bacteriological analysis negative), suggesting to the authors that CD4^pos^ cells were associated with low bacterial shedding, i.e., a more efficient immune response.

Several studies have dealt with *S. aureus* mastitis. Following infection by *S. aureus*, most of the lymphocytes that infiltrated the mammary tissue were located in close apposition to the alveolar and ductal epithelium (90). The comparatively low number of these cells in the alveolar lumen suggests that lymphocytes tend to remain associated with the epithelium. A preferential recruitment of CD4^pos^ cells over CD8^pos^ cells in milk was also reported in the course of experimentally induced infections with *S. aureus* (100). This was also true of chronic *S. aureus* infections, CD4^pos^ cells exceeding CD8^pos^ lymphocytes in milk, most of them expressing CD45RO, a marker of effector/memory cells (79). A 36-day follow-up study of experimentally induced *S. aureus* infection in lactating cows showed an inversion of the CD8/CD4 ratio during the initial acute clinical episode, then the proportion of CD4^pos^ cells decreased but remained higher than that of CD8^pos^ cells (101). A prominent feature was the progressive increase in the proportion of B cells, as observed in Riollet et al. (79). However, the preferential recruitment of CD4^pos^ cells during *S. aureus* mastitis has not been consistently reported (102). Cows resistant or susceptible to *S. aureus* mastitis differed in their CD4/CD8 ratio both in blood and in milk (103). The authors suggested that CD4 T cells were important in the resistance to *S. aureus* infection.

We can conclude from the available literature that all types of lymphocytes are recruited into the MG lumen during infection: CD4^pos^ and CD8^pos^ αβ T lymphocytes, γδ T cells, and few B lymphocytes. Most of the T lymphocytes in milk have an effector or memory (CD45RO^pos^) and activated (CD2^pos^ or ACT2^pos^) phenotype. Some of their functions have been identified, but we lack information on their antigen repertoire, and importantly, we need information on the functions of the T cells that populate the MG tissue. Recently, mucosal-associated invariant T cells (MAIT) have been found in human and bovine milk (104, 105). These cells recognize bacterial components through the interaction of their invariant TCR with the MHC-related protein MR1. They are preferentially found in the mucosae and are endowed with antibacterial properties (106). Bovine MAIT cells are recruited in milk of inflamed glands and they react to *E. coli* and *S. aureus* exposure by secreting IFN-γ and TNF-α, suggesting that they play an active role in the response to MG infection (105).

### The Known Functions of MG Lymphocytes

Earlier studies showed that MG mononuclear cells have a reduced capacity to proliferate in response to non-specific mitogens (Phytohemagglutinin, concanavalin A and pokeweed mitogen) when compared to blood lymphocytes (71, 102, 107), which could suggest that they are hyporesponsive. This is particularly true at parturition (108). However, milk cells produce IL-2, chemokines and IFNγ (107). Some reasons can be invoked to explain the reduced response to mitogens. Dry secretions and colostrum, but not milk, are able to inhibit the proliferative responses of peripheral blood mononuclear cells to mitogens (107). Milk CD8^pos^ T lymphocytes have been reported to have a suppressor function just after parturition compared to cytotoxic function in mid- and late lactation (109). The reduced proliferative response of milk CD4^pos^ T cells from MGs infected by *S. aureus* was attributed to the presence of suppressor effect of ACT2^pos^ CD8^pos^ T cells (102). Another explanation is that memory T cells, which are predominant in milk, tend to react less intensely to mitogens that do naïve T cells, which are predominant in blood (71). Little is known about the activities of MG lymphocytes. Milk CD2^pos^ lymphocytes have been shown to exert bactericidal and cytotoxic activities after stimulation with IL-2 (110). T cells from MG secretion incubated with autologous blood monocytes as APC proliferated in response to *S. uberis* exposure, irrespective of known previous MG infection by this pathogen (111). The T cells produced high levels of IFN-γ, and low levels of IL-10. A high proportion of responding cells were CD45RO^pos^. *In vitro* expanded cells with IL-2 were mostly CD8^pos^, released IFN-γ and some lineages could kill *S. uberis*.

Some information on the potential activities of milk lymphocytes was derived from transcriptomic studies. Milk cells from natural mammary infections expressed *IFNG* but not *IL2* or *IL4* mRNA (95). Milk cells from healthy cows in late lactation expressed *IFNG* mRNA but little *IL2* (112). Milk leukocytes from uninfected MGs did not express cytokine mRNA, whereas cells from *S. aureus* infected glands did: mRNA for IL-1α, IL1β, IL-6, TNF-α, IL-10, IL-12, but not IL-2 or IL-4 were found (79). Interesting information was derived from the measurement by RT-PCR of cytokine expression after stimulation with antibodies to CD3 or CD4^pos^, CD8^pos^ and γδ T cells isolated from milk and cisternal lavage of dry glands by magnetic cell sorting (113). CD4 and CD8 cells expressed mRNA for IFN-γ, IL-2, GM-CSF, TGF-β, and TNF-α. Expression was higher in cells from lactating than from dry MGs for CD4^pos^ T cells. γδ T cells substantially expressed mRNA of IFN-γ, GM-CSF, TGF-β, TNF-α, c-kit and IL-2R, but not mRNA of IL-2, IL-4, IL-6, IL-10, CD40L or FasL. Interestingly, c-kit encodes the stem cell factor (SCF) receptor, produced by mammary epithelial cells. As SCF acts synergistically with IL-2, IL-7 and IL-15 to induce proliferation and cytokine production by intraepithelial lymphocytes, c-kit production suggests a possible interaction between epithelial cells and γδ T cells in the bovine MG. CD4^pos^ and CD8^pos^ cells expressed TGF-β mRNA, but γδ T cells had the highest level of expression. The authors suggested that the activation of γδ T cells via the SCF receptor exerted a suppressive effect on the production of cytokines by other T cells during lactation.

Another source of information on the effector lymphocytes activated in or recruited to the MG has been gained from mouse models, keeping in mind that the translation to the MG of ruminants requires verification, due to differences relating among other things to development, involution, and link with the MALT. Investigations on healthy non-infected mouse MG have yielded results pertinent to the handling of antigens in the MG (56). Mammary DCs are able to process a protein antigen like ovalbumin and to cross-present it to lymphocytes. However, their limited expression of co-stimulatory molecules (CD80 and CD86) may give them the function of tolerogenic DCs. Moreover, during lactation and early involution, but not in fully involuted glands, there is a limited presentation of antigens (ovalbumin) to antigen-specific naïve T cells in the draining inguinal LN. Relatively few CD4^pos^ T cells were present in the lactating MG, but their numbers increased during the post-weaning involution. A proportion of these cells were RORγ T^pos^ (Th17 cells) during lactation, in increasing numbers at the beginning of involution, whereas FoxP3^pos^ T cells (Treg cells) were very few during lactation but increased markedly in numbers at the late phase of involution. Gata3^pos^ cells (Th2 lymphocytes), RORγT^pos^ Foxp3^pos^ (Th17-Treg lymphocytes), and RORγ T^pos^ Gata3^pos^ cells (Th17-Th2 lymphocytes) were also present. The authors concluded that some form of immune tolerance was present to prevent the lactating and involuting MG from developing self-reactive immunity against milk components. Mouse mastitis models were used to characterize the lymphocytes recruited in the MG by infection. Inoculation of *S. aureus* into the MG through the teat canal induced the recruitment of IL-17-producing γδ T cells, IL-17^pos^ (Th17) and IFN-γ^pos^ (Th1) lymphocytes, followed 5 days post-infection by an influx of CD4^pos^ CD25^pos^ (Treg) cells (114, 115). Similar observations were made after infection of the MG with *E. coli* (116). There was an influx of IL-17-producing cells into the MG as soon as 24 h post-infection, with a further increase at 48 h post-infection. The increases were due to γδ T cells and mainly to CD4^pos^ (Th17) cells. Of note, Th17 cells were also present in control glands, although in lower numbers than in inoculated glands, and IL-17 was detected, in accord with the reported influx of Th17 lymphocytes early in involuting glands after weaning (56), as the pups were removed from the mothers before inoculation. Neutralization of IL-17 with antibodies reduced the recruitment of neutrophils to the MG, strongly suggesting that IL-17-producing cells play a major role in the defense of the MG against infections (115, 116). Experiments with immunized mice are awaited to unravel the role of T cell immunity in the anamnestic response of the MG to infections. The relevance of the mouse mastitis model is supported by the observation that local immunization of the MG of cows during the dry period after priming by the intramuscular route elicited resident Th17 cells in the mammary tissue and an increased production of IL-17 in the tissue compared to unimmunized control glands upon infection challenge with *E. coli* (117). This immune response was associated with an improved control of infection (118).

### The Telltale Story of the Antigen-Specific Recruitment of Leukocytes to the MG

Early observations suggested that some form of hypersensitivity developed in the MG after an initial infection or exposure to bacteria. One month after an initial clinical infection by *Mycoplasma dispar*, when bacteria were no longer isolated and SCC had returned to normal, the inoculated quarter was able to eliminate the infection, contrary to the uninoculated quarters (5). In protected quarters, the influx of neutrophils was markedly amplified at the onset of infection. Milk leukocytosis and mastitis severity were intensified in response to intracisternal infusion of staphylococcal material (killed bacteria or cell wall extract) after a previous parenteral injection of heat-killed *S. aureus* (6). The authors concluded, “Cell-mediated immunity has a significant part in the pathogenesis of staphylococcal infection.” Thus, it appears that sensitization of the MG to bacterial antigens can either induce protection or aggravate the disease. The sensitization of the MG was not due to antibodies. The enhanced neutrophil influx in response to *S. aureus* infection in MG of ewes systemically immunized with live staphylococci was not transferred to non-immunized ewes by intramammary infusion of immune serum at the time of infection (50). Sensitization of cows to ovalbumin during lactation by subcutaneous injection of the antigen in incomplete Freund's adjuvant elicited a milk leukocytosis upon intracisternal infusion of soluble ovalbumin, which was not reproduced by infusion of antigen-antibody complexes (2). The demonstration of the role of lymphocytes in antigen-specific milk leukocytosis was obtained by adoptive transfer of cells from antigen-sensitized guinea pigs to naïve recipients (119, 120). The MG leukocytosis occurred only in animals that presented a delayed-type cutaneous hypersensitivity to the sensitizing antigen (119). In these experiments, most of the recruited cells were neutrophils, but macrophages and lymphocytes were also recruited, contrary to eosinophils. Interestingly, the influx of eosinophils into the MG lumen of heifers or sensitized ewes can be elicited by intraluminal infusion of nematode antigens (121–123). The route of immunization may be of consequence. When the antigen-induced MG response of guinea pigs was triggered with killed *S. aureus*, sensitization with this antigen by the intradermal route induced a stronger response than sensitization by the intramammary route, but when the intramammary challenge was with live *S. aureus*, milk leukocytosis occurred more rapidly in locally vaccinated animals and the severity of infection was less than with intradermal vaccination (124). The authors concluded that the intradermal route induced primarily a delayed type hypersensitivity but not an optimal protection. The magnitude of the milk leukocytosis and the cutaneous reaction to killed staphylococci were related, but the cellular composition was different: neutrophils dominated in milk whereas mononuclear cells were predominant in the skin at 24 h post-challenge.

The phenotype of the cells migrating to the MG lumen upon luminal challenge with antigen was investigated in cows sensitized by subcutaneous immunization with purified *S. aureus* α-toxin (125). Neutrophils represented the dominant cell type during the first 72 h post-challenge. Their expression of CD11b and CD18 was higher than on blood neutrophils, which attested a stimulated phenotype, supported by an efficient milk phagocytic bactericidal activity (126). CD8^pos^ T lymphocytes were recruited as soon as 12 h post-challenge, before CD4^pos^ T cells that peaked at 96 h. There was more N12^pos^ (WC1-) than WC1^pos^ γδ T cells in milk before and after challenge, but the proportion did not change after challenge. Most milk lymphocytes were CD45RO^pos^. Intriguingly, no TNF-α or IL-1β was detected in milk by ELISA, and there was very little increase in milk serumalbumin concentration even at peak leukocytosis, showing a disconnection between blood/milk barrier leakage and diapedesis and transepithelial migration of leukocytes. More recently, the enrichment of the bovine immunology toolbox enabled a more in-depth analysis. IL-17A and IFN-γ were detected at the onset of the neutrophilic inflammatory response in the milk of ovalbumin-induced immune response of sensitized cows, whereas IL-1β and IL-6 were found in only a few samples (127). The analysis by RT-qPCR of cytokine expression by the recruited milk cells showed that the genes encoding IL-17A, IL-17F, IL-21, IL-22 and IFN-γ were overexpressed with a peak at 8 h post-challenge, at the very beginning of the cell influx. These genes were also overexpressed in the tissue of the challenged compared to the control unchallenged glands. mRNAs encoding the chemokines CCL2, CCL5, CCL20 and CXCL10 were also overexpressed. Examination of the tissue of reactive MGs by immunohistochemistry revealed the presence of small numbers of IL-17A-reactive cells, most of them in subepithelial position or closely associated with the epithelial lining. Overall, these results suggest that type 3 and type 1 immunity are associated with the mammary antigen-specific immune response (mASR). This was confirmed by the finding that the mASR elicited following sensitization by the parenteral route correlates with the generation of circulating blood CD4^pos^ lymphocytes that produce IL-17A and IFN-γ (128). The protective role associated with the presence of Th17 cells in the MG (117) fits with these findings and the better protection against *E. coli* endowed by local immunization (118) is in accordance with the intramammary route for best protection as reported previously with *S. aureus* mastitis (124).

**Figure 2:**
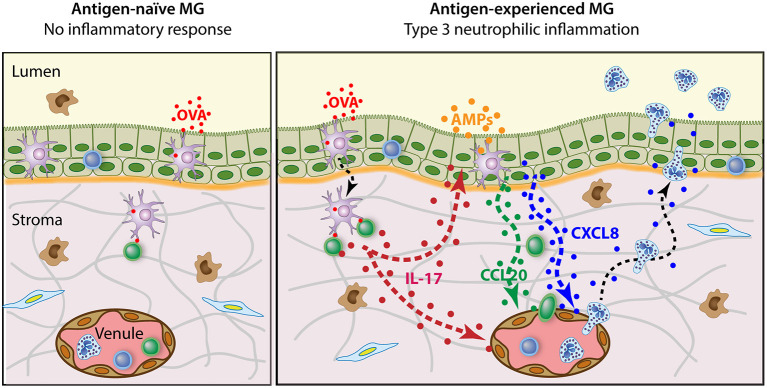
Schematic representation of the antigen-specific mammary inflammatory response. The antigen-naïve MG does not react to the intraluminal infusion of pyrogen-free antigen such as purified ovalbumin (OVA). By contrast, when OVA is infused in the lumen of antigen-experienced MGs, an intense neutrophilic inflammation develops. This reaction depends on the presence of Th17 cells in the mammary tissue and involves the local production of IL-17A and IL-17F. In turn, mammary epithelial and endothelial cells secrete chemokines such as CXCL8 and CCL20 that attract neutrophils and lymphocytes, and express adhesion molecules that facilitate diapedesis and transepithelial migration. Additionally, IL-17 in conjunction with pro-inflammatory cytokines induces mammary epithelial cells to produce antimicrobial peptides (AMPs).

The experiments that uncovered the antigen-specific MG immune response gave us revealing insight into the mechanisms of cell-mediated immunity in the udder of ruminants. They showed that the MG can be sensitized to antigens without local immunization (even though protection is better with local infusion of antigen), and during lactation (whereas local immunization is efficient during the dry period). The response is systemic, including the skin and the MG. The mammary response can be induced in lactating MGs with small amounts of antigens that are inert in the MG of naïve animals. This means that recognition by the innate immune system of the MG is dispensable to trigger the reaction. As epithelial cell junctions are tight during lactation, this supposes that the luminal antigen is taken up by APCs able to sample the lumen, processed, and presented to antigen-specific lymphocytes. These antigens trigger the response in the absence of MAMPs, which could stimulate the APCs. The reaction of lymphocytes in the absence of strong co-stimulation by APCs supposes that these responding lymphocytes are not naïve. The promptitude of the milk leukocytosis and absence of pre-existing inflammation indicate that these lymphocytes are present in the mammary tissue before antigen exposure. The type of response (neutrophilic inflammation) strongly suggests that these memory lymphocytes are effectors of the type 3 immunity. The inferred sequence of events is schematized in Figure 2.

## A Current Vision of Mg Cell-Mediated Adaptive Immunity and Implications for Vaccination

The current model of adaptive immunosurveillance proposes that several memory T cell subsets perform distinct roles. These subsets are defined based first on trafficking patterns, and second on other features such as cytokine, surface markers, and transcriptional signatures (27, 129). Two main groups are distinguished: the Trm cells that establish long-term residence in non-lymphoid tissues and are frontline responders, and the circulating memory T cells that traffic between the blood and secondary lymphoid organs and can be recruited to non-lymphoid tissues in case of inflammation. This distinction is not absolute, because some Tcirm may patrol peripheral tissues, and some Trm may recirculate. It is also clear that there are a number of subsets in each group. A subset of Tcirm, termed central memory T cells (Tcm), have a high proliferative potential and generate cohorts of antigen-specific effector T cells when reactivated, but do not patrol non-lymphoid organs. Another subset, the effector memory T cells (Tem), has limited expansion potential but can rapidly turn into effector T cells with a high degree of functionality upon antigenic encounter. Another subset of Tcirm, peripheral memory cells (Tpm), can recirculate between lymphoid and non-lymphoid tissues and contributes to the surveillance of body tissues (130). Trm differentiation is at least partially executed after migration to non-lymphoid tissues, and can thus be conditioned there (28). These processes ensure a greatly enhanced adaptive immune response upon reinfection.

Most of the studies that defined memory T cell subsets dealt with CD8+ T cells. There are differences in the migration patterns of CD4 and CD8 memory T cells to different compartments of barrier tissues. The CD8^pos^ Trm are found primarily in epithelial layers, while CD4^pos^ Trm are found in the lamina propria and parenchyma (131, 132). We have seen that this hold true with the MG. After arriving in their tissue of destination, Trm cells remain in situ for long periods. A number of chemokines and their receptors, or integrins and their matrix or epithelial cell ligands, have been incriminated in the attraction and retention of Trm cells (132). Their importance varies according to the tissue, and none seems to be an absolute requisite. We lack knowledge about the local molecular microenvironment that makes mammary tissue special for lymphocytes. The cytokine TGF-β has been shown to contribute to CD8 terminal differentiation and intra-epithelial maintenance through induction of CD103 (integrin αE interacting with the epithelial E-cadherin) in mice intestine (133, 134). Another possible survival and retention signal shared by several epithelia is the production of serum amyloid A (SAA). SAA is produced constitutively by the MG, in particular at the duct level (135). Porcine milk SAA is a chemoattractant for sow B lymphoblasts (136). Also, SAA promotes local Th17 responses (137). A signal specific to the MG might be casein. Casein has been found in the macrophages that populate the MG epithelium (49), and has been shown to induce monocyte differentiation and production of GM-CSF (138). The chemokine CXCL3 is produced constitutively by MECs (139) and could play a role in the attraction of patrolling cells that express the cognate receptor CXCR2. Much remains to be learned about the homing and retention of Trm cells in the MG.

Owing to their location, Trm cells are at the frontline to perform tissue immunosurveillance. Trm cells are not immobile. Both intraepithelial and stromal cells can move, although slowly, and random migration along with cytoplasmic extensions allow them to scan their environment, establishing contact with epithelial and stromal cells (66). In the MG, they are bound to contact and interact with the network of ductal macrophages. Trm cells trigger protective innate and adaptive immune responses. Upon antigen re-exposure, memory T cells turn into effector memory T cells with a high degree of functionality. These processes ensure a greatly enhanced adaptive immune response upon reinfection.

**Figure 3:**
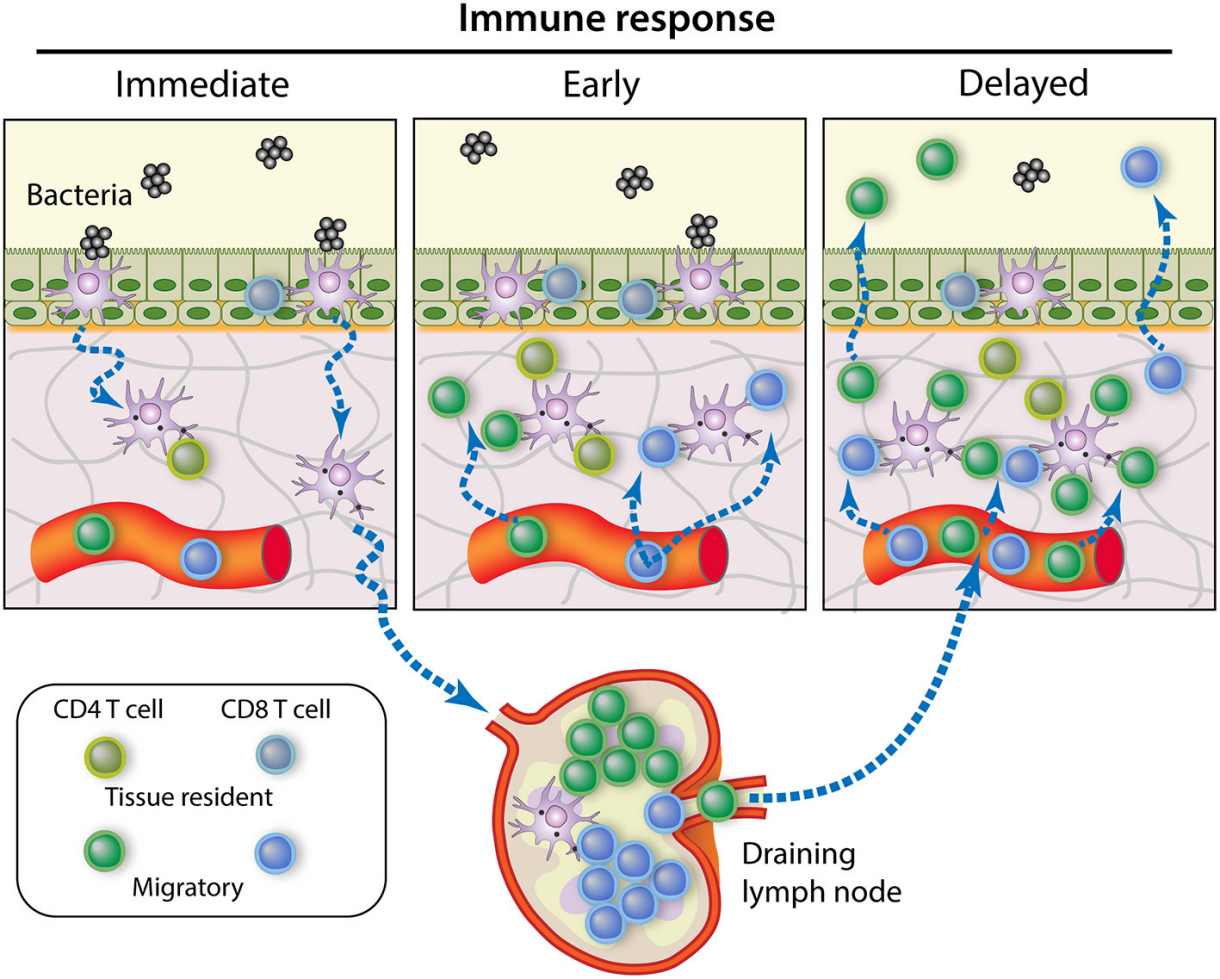
Contribution of memory T cell subsets to the control of MG infection. Immediate response to pathogen intrusion is achieved by memory T cells present in the tissue at the onset of infection, namely the resident memory T cells. If the infection is not cleared at this stage, the early local inflammation recruits circulating memory T cells that amplify the immune response. Meanwhile, bacterial antigens are presented by APCs migrating from the infected tissue to the draining lymph nodes, where central memory T cells begin to proliferate and generate new effector T cells. After a few days, these effector T cells will migrate to the site of infection in great numbers, making a delayed but robust contribution to pathogen control [Modified from (144)].

The modes of action of Trm cells are potentially diverse, and much remains to be discovered on this subject. CD8^pos^ Trm could perform cytotoxic or suppressive activities, but they can also operate through cytokine (IFNγ, TNF-α) secretion, activation of DCs and recruitment of B and T cells (140). There is now evidence for the role of CD4^pos^ Trm in protection against pathogens in multiple epithelial barrier sites (141). CD4^pos^ Trm exhibit rapid recall function and can produce cytokines such as IFN-γ and IL-17, triggering chemokine production and broad immune activation, orchestrating local recall responses (142). Resident memory lymphocytes can play a prominent role not only in immediate response to infection, but they are also particularly adept at controlling latent and persistent infections (143). A main asset of Trm cells is that they are in a position to intercept the pathogens at the very beginning of infection (29). They can react as soon as APCs such as ductal macrophages have processed and presented the cognate antigens to the Trm and patrolling Tcm cells present in the epithelium and the lamina propria. Later on, accumulation of recirculating memory T cells and finally the wave of the secondary effector lymphocytes give full magnitude to the adaptive immune response (Figure 3).

Now we can postulate how MG immunity fits into current concepts of peripheral tissue monitoring by memory T cells and draw implications for an effective vaccination. The neutrophilic inflammation induced by luminal infusion of antigen into the MG of sensitized animals can be explained by the induction of Trm cells in the epithelium and sub-epithelial locations. Systemic immunization elicits the antigen-specific mammary response because circulating precursors of memory T cells visited the MG and matured there into Trm cells. However, local (intramammary) immunization is more efficient because the local inflammation will attract more circulating T cells than in non-infused uninflamed glands. Transepithelial antigen exposure is more efficient than interstitial antigen exposure because the antigen is taken over by epithelial or sub-epithelial APCs that are conditioned by the mammary microenvironment. In turn, the mammary APCs will instruct the antigen-specific naïve T cells encountered in the MG draining lymph nodes. This will orient the development of the CD4^pos^ and CD8^pos^ T cells both in terms of effector/memory, recirculation/location, and immune types (129, 132).

**Figure 4:**
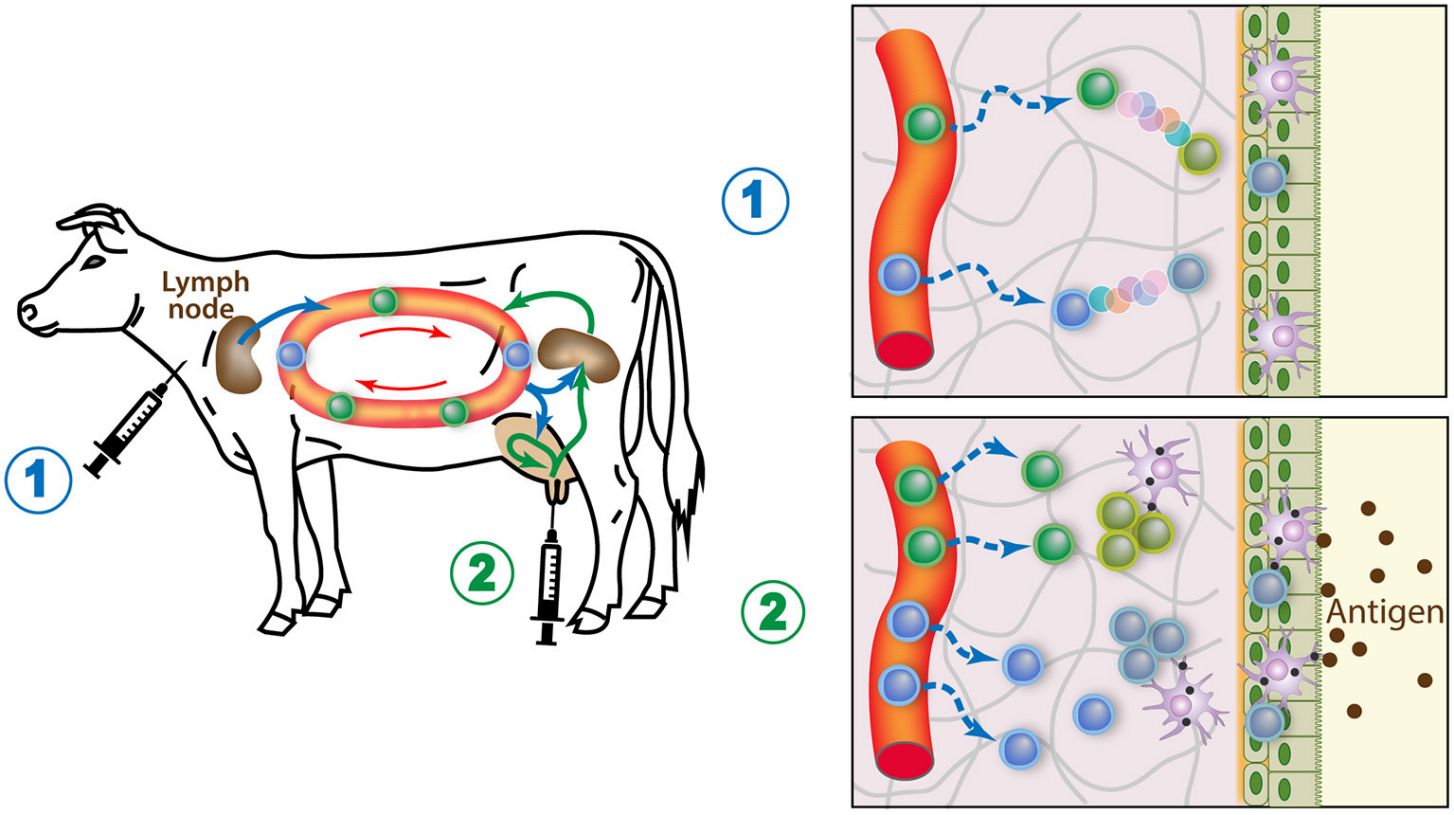
How the prime and pull vaccine strategy would populate the MG with memory lymphocytes. 1) A parenteral immunization (prime) elicits circulating antigen-specific T cells, some of which seed the MG tissue and mature into resident memory T cells. 2) A booster injection of antigen in the gland lumen causes local APCs to stimulate the resident memory T cells cells, which in turn induces an enhanced recruitment of circulating T cells, boosts the systemic immune response, and induces replication of the local Trm cells. These responses maximize the generation of local resident memory T cells.

Local immunization is more efficient in dry than in lactating period because the epithelium is more permeable, and more APCs and lymphocytes are present in the mammary tissue. The recurring observation that the best way to induce a local immune response is the combination of systemic immunization followed by local intramammary infusion of antigen can be explained if we consider that memory T cells achieve their maturation in their destination tissue and can multiply upon antigen-re-exposure (28). The antigen-experienced lymphocytes elicited by the primary systemic immunization circulate in the body, including the MG. The inflammation caused by the antigen boost (resulting from the use of whole bacteria or MAMPs) will attract more lymphocytes. Inflammation recruits effector T cells regardless of antigen specificity, but the antigen-specific lymphocytes will be retained and induced to replicate before maturing into resident memory cells (145). Both CD4^pos^ and CD8^pos^ Trm cells could be elicited, as cross-presentation by mucosal APCs might present soluble antigens through MHC class I (146). As a result, many more antigen-specific Trm cells will populate the locally vaccinated gland. Due to the importance of local tissue imprinting by adjuvant-induced inflammation and antigen-dependent replication of memory T cells, all glands will have to receive the vaccine. The time-window most favorable to maximizing the magnitude of the immune response, when memory precursors circulate, is likely to be an important parameter of success. A new strategy for generating Trm cells in the peripheral tissues involves a “prime and pull” protocol in which parenteral vaccination (prime) is followed by the recruitment and activation of antigen-experienced T cells with local application of vaccine (pull) (Figure 4). This approach has been used to immunize cows against *E. coli* (118). The combination of systemic and local routes of immunization induced a protective immune response involving Th17 cells in the tissue of locally immunized MG (117).

## Conclusions and Recommendations

It follows from many studies that the MG is a site of induction of local and systemic immunity that can be activated with best efficacy during or after MG involution. The gut-MG axis is not operational in dairy ruminants, and the MG does not belong to the CMIS. It does not accommodate organized lymphoid formations, except a few lymphocyte aggregates that appear during its development or in the course of chronic infections. However, the epithelium of healthy MGs is populated with many macrophages, a few CD8 T cells, perhaps a few DCs, while the connective subepithelial tissue contains a few CD4 T cells. The MG can be seeded with antigen-experienced T cells by appropriate vaccination strategies. Due to the crucial importance for the outcome of infection of a swift immune reaction to intrusion of pathogens into the MG lumen, induction of resident memory T cells is the best way to enhance the epithelial barrier and neutrophilic inflammation efficacy. This will control infection before bacteria establish a persistent infection.

In spite of the many studies and recent advances in MG immunity, there is still a lot to be discovered before we can rationally design mastitis vaccines (147). Progress in understanding the mechanisms underlying the generation, maintenance, and function of memory T cells in the MG will be essential. One implication of lymphocyte tissue residence is that most of the MG adaptive immune system remains hidden from view if we restrict studies to easily accessible milk cells. Using the induction of Trm cells as correlate of protection is loaded with difficulties linked to small numbers, heterogeneity and difficulty to collect them in sufficient numbers without activation or damage (66). Yet, we lack reliable correlates of protection for mastitis vaccines (148). Besides the relative proportions of T lymphocyte subpopulation phenotypes, we have little information on their functions, particularly on the cells that populate the MG tissue. We have little information on the functions of the intraepithelial CD8^pos^ T cells (αβ and γδ T cells) that are at the frontline of MG infections. The same question applies to the role of ductal macrophages during infection or local immunization. The phenotype of milk and mammary tissue lymphocytes and APCs deserves better characterization with the newly available reagents. Above all, the functional capacity of mammary lymphocytes need to be investigated and compared to their blood counterparts. Gaps in our knowledge of adaptive immunity to the MG are numerous (Table 2), filling them would greatly assist researchers in designing novel mastitis vaccines.

**Table 2:** Knowledge gaps that hinder the design of efficacious mastitis vaccines.

Molecular determinants of the homing of lymphocytes to the MG of ruminants
Nature of the vascular addressins and chemokines
Nature of lymphocytic integrins and chemokine receptors
Detailed phenotype of lymphocytes that populate the mammary tissue
In healthy MG
During and after infection
In immunized MGs
Nature and functions of the APCs that condition the immune response to luminal antigens
Role of ductal macrophages in the sensing of bacteria
Role of stromal macrophages
Nature of the tissue factors that condition effector and memory T cell differentiation and maintenance *in situ*
Functions of tissue and milk lymphocytes
Nature of the interactions of Trm cells with mammary epithelial cells and ductal macrophages
Roles of *γδ* T cells in adaptive immunity
Roles of intraepithelial CD8^pos^ cells in the defense of the MG epithelium

The orientation to be given to the immune response for the best possible protection is still open to question, even though arguments in favor of Th17 cells and type 3 immunity for the defense of the MG against infections have been put forward (149). Several other questions call for an answer. How does the balance between adaptive pro-inflammatory and regulatory immunity should be tipped? This is a key point to avoid increasing the damages caused by the pathogen and the accompanying inflammatory response. How long is the long-lived epithelial immunity conferred by Trm cells in the MG? This will determine the recurrence interval for vaccination boosters. Does the shedding of lymphocytes (CD45RO^pos^) in milk corresponds to a physical loss of memory as it equates to a loss of MG memory T cells, which would result in short-lived adaptive immunity in the MG? The new powerful tools that are available to investigate the immune pathways and mechanisms make the search for answers to these questions feasible. Based on current knowledge, rekindling research on the immune cells that populate the healthy, infected, or immunized MG appears to be a most promising approach for the design of efficacious mastitis vaccines. However, this requires the mobilization of significant resources that are not easy to implement in the field of mastitis research. It is regrettable that during the last decades the funding of research on mammary immunity has been so parsimonious, which has discouraged researchers and hindered collaborations. We hope that this enticing field of research will attract new researchers and funding.

## Author Contributions

PR, GF, and RPM: conceptualization, writing-review and editing, and funding acquisition. PR: writing-original draft preparation. All authors contributed to the article and approved the submitted version.

## Funding

This work was supported by Agence Nationale de la Recherche (ANR-20-CE20-0023); INRAE.

## Conflict of Interest

The authors declare that the research was conducted in the absence of any commercial or financial relationships that could be construed as a potential conflict of interest.

## Publisher's Note

All claims expressed in this article are solely those of the authors and do not necessarily represent those of their affiliated organizations, or those of the publisher, the editors and the reviewers. Any product that may be evaluated in this article, or claim that may be made by its manufacturer, is not guaranteed or endorsed by the publisher.
